# Association of Soil Aggregation with the Distribution and Quality of Organic Carbon in Soil along an Elevation Gradient on Wuyi Mountain in China

**DOI:** 10.1371/journal.pone.0150898

**Published:** 2016-03-10

**Authors:** Liguang Li, Jason Vogel, Zhenli He, Xiaoming Zou, Honghua Ruan, Wei Huang, Jiashe Wang, Thomas S. Bianchi

**Affiliations:** 1 College of Biology and the Environment, Co-Innovation Center for Sustainable Forestry in Southern China, Nanjing Forestry University, Nanjing, 210037, China; 2 University of Florida, Institute of Food and Agricultural Sciences, Indian River Research and Education Center, Fort Pierce, Florida, 34945, United States of America; 3 Department of Ecosystem Science and Management, Texas A&M University, 2126 TAMU, College Station, Texas, 77843–2126, United States of America; 4 Institute for Tropical Ecosystem Studies, University of Puerto Rico, P. O. Box 70377, San Juan, Puerto Rico, 00936–8377, United States of America; 5 Administrative Bureau of Wuyishan National Nature Reserve, Wuyishan, Fujian, 354300, China; 6 Department of Geological Sciences, University of Florida, Gainesville, Florida, 32611, United States of America; Chinese Academy of Sciences, CHINA

## Abstract

Forest soils play a critical role in the sequestration of atmospheric CO_2_ and subsequent attenuation of global warming. The nature and properties of organic matter in soils have an influence on the sequestration of carbon. In this study, soils were collected from representative forestlands, including a subtropical evergreen broad-leaved forest (EBF), a coniferous forest (CF), a subalpine dwarf forest (DF), and alpine meadow (AM) along an elevation gradient on Wuyi Mountain, which is located in a subtropical area of southeastern China. These soil samples were analyzed in the laboratory to examine the distribution and speciation of organic carbon (OC) within different size fractions of water-stable soil aggregates, and subsequently to determine effects on carbon sequestration. Soil aggregation rate increased with increasing elevation. Soil aggregation rate, rather than soil temperature, moisture or clay content, showed the strongest correlation with OC in bulk soil, indicating soil structure was the critical factor in carbon sequestration of Wuyi Mountain. The content of coarse particulate organic matter fraction, rather than the silt and clay particles, represented OC stock in bulk soil and different soil aggregate fractions. With increasing soil aggregation rate, more carbon was accumulated within the macroaggregates, particularly within the coarse particulate organic matter fraction (250–2000 μm), rather than within the microaggregates (53–250μm) or silt and clay particles (< 53μm). In consideration of the high instability of macroaggregates and the liability of SOC within them, further research is needed to verify whether highly-aggregated soils at higher altitudes are more likely to lose SOC under warmer conditions.

## Introduction

On a global scale, soil organic carbon (SOC) reservoir is approximately twice as large as that of the atmosphere, and approximately three times that resides within the vegetation[1]. Even subtle changes in the overall mass of global SOC pools could trigger immense fluctuation in the concentration of CO_2_ in the ambient atmosphere. One potential modification of the SOC pool may occur due to the changes in microbial decomposition of organic matter in the soil. The stability of SOC against microbial degradation is thought to be contingent on various interactions between SOC chemistry, soil climate, soil fauna, and soil structure [2]. Of these factors, the effects of soil structure and/or aggregation on SOC dynamics remain one of the least understood, particularly in natural ecosystems. Water-stable aggregation rate has been recognized as one of the standard features of soil quality that affects SOC dynamics [3–7]. For example, the hierarchical structural organization of soil, which is typically expressed as the mean weight diameter of soil aggregates [8], may influence the temperature sensitivities that are associated with decomposition [2]. In agricultural systems, the sequestration or release of SOC is largely determined by the distribution of SOC within soil aggregates [3]. The mean residence time of organic carbon (based on ^14^C measurements) decreases in the order of aggregate-occluded C > mineral C > free C within California conifer forest soils, which suggests a significant role of aggregate protection in control of C turnover in soils [9].

Globally,~70%-73% of SOC resides within forest soils [10], thus, terrestrial ecosystem carbon storage, and in turn, global carbon balance is directly influenced by the accumulation and decomposition of organic carbon in the forest soils [11]. The interactions between temperature driven changes in SOC and soil aggregation comprise an element of uncertainty, with major consequences for current models of carbon turnover in soils. Hence, this issue is a matter of intense debate as relates to global climate change [12]. Soil organic matter may be protected from temperature fluctuations via microaggregation (53–250 μm) within macroaggregates (250–2000 μm), physical binding with soil clay and silt particles, and the biochemical formation of recalcitrant SOC compounds [13]. However, these processes remain to be fully understood.

Mountainous areas are known to be especially vulnerable to climatic change[14], where variations in altitude driven temperatures serve as a proxy for the effects of temperature on SOC accumulation under natural conditions. As altitude increases, mean annual precipitation (MAP) rises, while temperature decreases. This leads to the generation of different vegetation types, increased soil moisture, and lower soil temperatures. As a consequence, a larger volume of SOC is stored within the soils along elevation gradients [15, 16]. The objectives of this study were to: (1) quantify SOC in different size fractions of soil aggregates in soil samples collected from representative forestlands with representative vegetation along the altitude gradient of Wuyi Mountain, in southeastern China; and (2) examine the effects of temperature and soil aggregation on SOC distribution within different soil fractions.

## Materials and Methods

### Site Description and Experimental Design

Soil samples were collected from Wuyi Mountain National Nature Reserve (27°33'–27°54'N, 117°27'– 117°51'E) (Jiashe Wang, the authority responsible for this national nature reserve, issued the permission for each location of this study), which is located in the subtropical zone of southeastern China. They were collected from four forestland sites, i.e. a subtropical evergreen broad-leaved forest (EBF) at 690 m above sea level (ASL), a sub-alpine coniferous forest (CF) at 1140 m ASL, a subalpine dwarf forest (DF) at 1750 ASL, and an alpine meadow (AM) at 2060 ASL) within distinct vertical zones of vegetation along an elevation gradient on Wuyi Mountain. The age of the EBF is from 60 to 70 years, and the dominant species are *Castanopsis carlesii*, *Castanopsis eyrei*, and *Schima*, with a litter layer depth of 3–4 cm. The CF is a natural unevenly aged forest with an average age of 70 years, and dominant species comprising *Pinus taiwanensis*, *Oligostachyum oedognatum* and *Cunninghamia lanceolata*, with a litter depth of 5–7 cm. The DF is a mature forest of >100 years, with the dominant species *Symplocos paniculata*, *Tsuga heterophylla* and *Stewartia sinensis*, and a litter depth of 3–4 cm. The dominant species of AM are *Calamagrostis brachytricha*, *Rhododendron fortune* and *Mahonia fotrunei*, with the depth of wilted grass at 1–2 cm. pH of soils in this region is 4.54~5.22 [17]. These sites had been previously described [17, 18], and additional information is presented in Table 1.

**Table 1:** Altitude, mean annual precipitation (MAP), mean annual temperature (MAT), litterfall biomass (LB), soil type, bulk density (BD), soil texture, soil temperature (Tsoil) and soil moisture (Msoil) in evergreen broad-leaf forest (EBF), coniferous forest (CF), sub-alpine dwarf forest (DF) and alpine meadow (AM) soil along the gradient elevation of Wuyi Mountain, China (mean ± standard error, n = 4).

Vegetation	Altitude	MAP^a^	MAT^a^	LB^b^	Soil Type^c^	Depth	BD^d^	Soil texture (%)^d^			Tsoil	Msoil
	(m)	(mm)	(℃)	(Kg m^-2^ y^-1^)		(cm)	(mg m^-3^)	Sand	Silt	Clay	(℃)	(%)
EBF	690	1700	17–19	0.32	Typic	0–10	0.87	68.8	20.0	11.2	15.8±0.2	26.6±1.3
					Haplohumults	10–25	0.91	61.9	22.8	15.3	15.9 ±0.4	21.4±3.1
CF	1140	2000	14.5	0.30	Typic	0–10	0.64	61.9	28.1	10.0	14.3 ±0.4	33.4±3.2
					Palehumults	10–25	0.80	59.7	20.7	19.6	14.4 ±0.5	31.5±2.4
DF	1750	2200	11.2	0.19	Typic	0–10	0.61	58.0	30.1	11.9	12.3 ±0.6	44.3±1.9
					Dystrudepts	10–25	0.80	51.4	32.2	16.3	11.6 ±0.4	43.7±5.3
AM	2060	3100	9.1	0.07	Histic	0–10	0.54	52.1	34.1	13.8	11.8 ±0.6	59.9±3.2
					Humuaquepts	10–25	0.70	46.2	39.1	14.7	11.2 ±0.8	54.0±0.9

^a^He, Lan [48]

^b^He, Wang [49]

^c^Chen [50]

^d^Bu, Ruan [7]

We established four square 3 m replicate plots for soil sampling at each of the four field sites. Soil samples were collected at the depths of 0–10 cm and 10–25 cm, with a core sampler (Ø3 cm), from each plot in September 2010. Each plot soil sample consisted of a composite of 8–10 cores, randomly collected within each plot.

### Aggregate-size fractions

Before wet sieving, all field-moist soil samples were passed through a 2000-μm sieve and air dried. Subsamples of air-dried soil (100 g each) were placed on a 2000-μm sieve and submerged in deionized water for 5 min., while alternately (manually) raising and lowering the sieve by 3 cm, according to the methods of Six, Callewaert [4]. The organic material that remained floating in water above the 2000-μm sieve was removed following a 2-min. cycle. The fraction that remained on the 2000-μm sieve was defined as macroaggregates, which were collected in an aluminum pan and oven dried. The water and soil that passed through the 2000-μm sieve was poured onto a 53-μm sieve, where the sieving process was repeated. Subsequently, all fractions were gently collected into an aluminum pan and oven dried (50°C), following the careful removal of all gravel and stones. The weights of all the fractions were recorded. The sand content of all aggregate fractions was determined, and the aggregate weight percentage was corrected via the following formula:

$$\text{ Aggregate weight \% }\left({\text{w}}_{\text{i}}\right)=\;\frac{\text{total fraction weight}-\text{same sized sand weight in the fraction}}{\sum \text{sand corrected weight}}$$ (1)

Macroaggregates were further separated following the methods of Six, Callewaert[4]. Subsamples (6g each) of the oven-dried macroaggregates were slaked in deionized water for 20 min, and then placed on a modified 250-μm sieve along with 50 glass beads (Ø4 mm). The soil and glass beads were kept submerged and agitated on a reciprocal shaker until all the macroaggregates were dissolved (5–10 min). A constant flow of water was applied to ensure that the microaggregates, as well as other materials released from the broken macroaggregates, quickly passed through the 250-μm sieve to avoid further disruption. The soil that passed through the 250-μm sieve was then transferred to a 53-μm sieve and sieved for 2 min. as described above, yielding a total of three fractions isolated from the macroaggregates: coarse particulate organic matter (>250 μm; cPOM), microaggregates within macroaggregates (53–250 μm; MM) and macroaggregate occluded silt and clay particles (<53 μm, MSC). All fractions were subsequently dried at 60°C and weighed to determine the proportion of each aggregate size in a soil sample, and then ground for subsequent analysis.

The statistical index of aggregation is expressed as mean weight diameter (MWD), the average size of soil aggregates, which was calculated according to the following equation [4]:

$$\text{MWD}=\overset{n}{\underset{i=1}{\sum }}\bar{x_{i}}w_{i}$$ (2)

Where MWD equals the sum of products of the mean diameter of each size fraction ($\bar{x_{i}}$) and the proportional weight of the corresponding size fraction (w_*i*_). The mean diameter of the largest fraction was 2000 μm.

### Soil Analysis

Each of the soil samples prepared for SOC and N analyzes weighed 20 ~30 mg. The organic carbon and total nitrogen in the soil aggregates and bulk soil were measured via a dry combustion technique using a C/N/S-Analyzer (Vario EL III, Elementar, Germany). Soil temperatures were measured by Watchdog weather stations (Spectrum Technologies, Inc., IL, USA) in each subplot at the 5 and 15 cm depth respectively during sample collection. Soil moisture was determined as the difference between field moist and dried (24h at 105°C) soil weight. Soil bulk density was determined by the core method [7]. The particle-size was determined by wet sieving and sedimentation using the pipette sampling technique [7].

The percentage of aggregated organic carbon to total organic carbon (TOC) was estimated following the method of Pulleman, Six [6]:

$${\text{P}}_{\text{f}}\left(\text{\% of total organic carbon}\right)=\frac{\text{OC }\left(\text{f}\right)\left({\text{mg g}}^{-1}\text{ fraction}\right)\times {\text{w}}_{\text{i}}}{\text{total organic carbon }\left({\text{mg g}}^{-1}\right)}$$ (3)

Where OC (f) is the organic carbon concentration in the corresponding aggregates and w_i_ is the proportional weight of the corresponding size fraction.

### Statistical Analysis

One-way ANOVA followed by an LSD test was used to determine whether the differences in distribution of different soil aggregate fractions, MWD, and soil organic carbon from the same vegetation soil. It was also used to determine whether the differences in C:N ratios and organic carbon distribution among soil aggregates were significant at the same soil depth for the same vegetation. The statistical significance for all tests was set at *P*<0.05. Pearson correlation coefficient analysis was performed to reveal the relationship between SOC in bulk soil, the distribution of SOC within different size fractions of soil aggregate, soil clay content, soil moisture, soil silt and clay content, soil coarse particulate organic matter content, soil temperature, and MWD. All statistical analyses were performed using PASW Statistics 18.0 (USA) and Microsoft Excel 2007 software.

## Results

### Water-Stable Aggregate Distribution

The distribution of water-stable aggregates in soils varied with vegetation types (Table 2). At the 0–10 cm depth, coarse particulate organic matter increased with altitude; however, the percentage of microaggregates decreased with altitude. The macroaggregate occluded silt and clay fractions were lowest in EBF soil, whereas silt and clay particles were lowest in AM soil. At the 10–25 cm depth, the coarse particulate organic matter was highest in AM soil. The macroaggregate occluded silt and clay particulates were also lowest in EBF soil; the microaggregates and silt and clay particles were highest in CF soil.

**Table 2:** Distribution of coarse particulate organic matter (cPOM), microaggregates within the macroaggregates (mM), macroaggregate occluded in silt and clay particles (scM), microaggregates (Micros) and silt and clay particles (SC), mean weight diameter (MWD) and soil organic carbon in soil samples collected at evergreen broad-leaf forest (EBF), coniferous forest (CF), sub-alpine dwarf forest (DF) and alpine meadow (AM) on Wuyi Mountain, China (mean ± standard error, n = 4).

Depth (cm)	Vegetation	cPOM (%)	mM (%)	scM (%)	Micros (%)	SC (%)	MWD (μm)	SOC (mg g^-1^)
0–10	EBF	39±7c	8±2c	10±3b	36±1a	7±1ab	700±10c	36.2±3.5c
	CF	40±2c	7±1c	14±1ab	25±5b	13±5a	730±60c	42.4±6.4bc
	DF	53±1b	12±1b	12±2b	17±4c	5±3b	900±20b	58.4±4.6b
	AM	59±3a	17±1a	18±3a	3±3d	4±2b	1050±10a	134±12a
10–25	EBF	38±3b	9±1c	11±4c	34±3a	8±1c	710±40b	22.5±7.7c
	CF	27±2c	7±1c	19±3b	27±4b	20±3a	640±70b	28.6±7.3c
	DF	34±8bc	10±4b	17±6b	27±6b	12±4b	730±90b	39.8±1.8b
	AM	44±1a	16±1a	26±1a	7±7c	7±5c	980±20a	93.8±8.2a

Different letters within the same row under each depth indicate significant differences among the vegetation types at P<0.05 (n = 4).

At the 0–10 cm depth, soil aggregation rate, MWD, increased with altitude. At the 10–25 cm depth, MWD was highest in AM soil. (Table 2).

### Organic Carbon in Water-Stable Aggregates

The concentration of SOC in soil aggregates and bulk soil increased with increasing elevation (Table 3). For the same vegetation type, SOC concentrations varied significantly among different size fractions of soil aggregates. At the 0–10 cm depth, EBF, CF, and DF soils had the lowest SOC concentration within the coarse particulate organic matter fraction, while the AM soil had the lowest SOC content in macroaggregate occluded silt and clay particles. At the 10–25 cm depth, EBF soil had the lowest SOC concentration within the coarse particulate organic matter fraction, while SOC concentration in CF soil was not significantly different among the different size fractions of soil aggregates.

**Table 3:** SOC concentrations and carbon to nitrogen (C: N) ratios in coarse particulate organic matter (cPOM), microaggregates within the macroaggregates (mM), macroaggregate occluded silt and clay particles (scM), microaggregates and silt and clay particles of samples collected at an evergreen broad-leaf forest (EBF), coniferous forest (CF), sub-alpine dwarf forest (DF) and alpine meadow (AM) on Wuyi Mountain, China (mean ± standard error, n = 4).

Depth	Vegetation	SOC in soil fractions (mg g^-1^)					C:N in soil fractions				
(cm)		cPOM	mM	scM	Micro	SC	cPOM	mM	scM	Micro	SC
0–10	EBF	22.4 ±8.2c	44.9 ±5.7b	59.6 ±5.6a	45.4 ±2.5b	59.2 ±10.2a	14.4±0.7	14.2±0.5	13.6±0.4	13.4±0.3	13.0±0.4
	CF	25.2 ±10.2c	46.6 ±1.9b	54.9 ±3.9ab	58.4 ±0.3a	50.6 ±2.0ab	18.5±0.5a	16.1±0.6ab	13.7±0.2ab	15.0±0.4ab	13.1±0.6b
	DF	48.7 ±9.1b	70.0 ±6.3a	66.7 ±4.8a	74.2 ±3.9a	75.8 ±2.9a	12.7±0.5a	11.8±0.1ab	11.0±0.2b	11.8±0.2ab	11.1±0.5b
	AM	132 ±12.7ab	148±24.4a	120 ±0.1b	130 ±1.1ab	120 ±19.5b	14.9±0.1	15.0±0.1a	14.7±0.4	15.4±0.2	15.4±0.1
10–25	EBF	12.4 ±4.9c	26.1 ±4.4b	30.4 ±1.6a	27.2 ±5.4b	38.5 ±3.4a	13.8±0.2	13.0±0.8	12.7±0.6	12.1±0.1	12.1±0.2
	CF	30.8 ±0.5	29.4 ±5.2	25.4 ±5.4	27.5 ±8.3	23.4 ±4.8	18.4±0.4a	15.7±0.1ab	13.0±0.3b	13.2±0.1ab	11.5±0.6b
	DF	35.9 ±6.1b	43.4 ±3.5ab	38.6 ±0.8ab	43.7 ±5.7a	38.2±5.3ab	11.0±0.3	10.9±0.5	10.5±0.2	11.0±0.2	10.6±0.4
	AM	91.9 ±16.6ab	109 ±16.3a	90.9 ±10.1ab	97.8 ±27.7ab	83.8 ±12.3b	14.9±0.1	14.8±0.3	14.5±0.2	14.9±0.2	15.0±0.1

Values followed by different letters within the same row under each depth are significantly different among different soil aggregate fractions at *P*<0.05.

**Fig 1:**
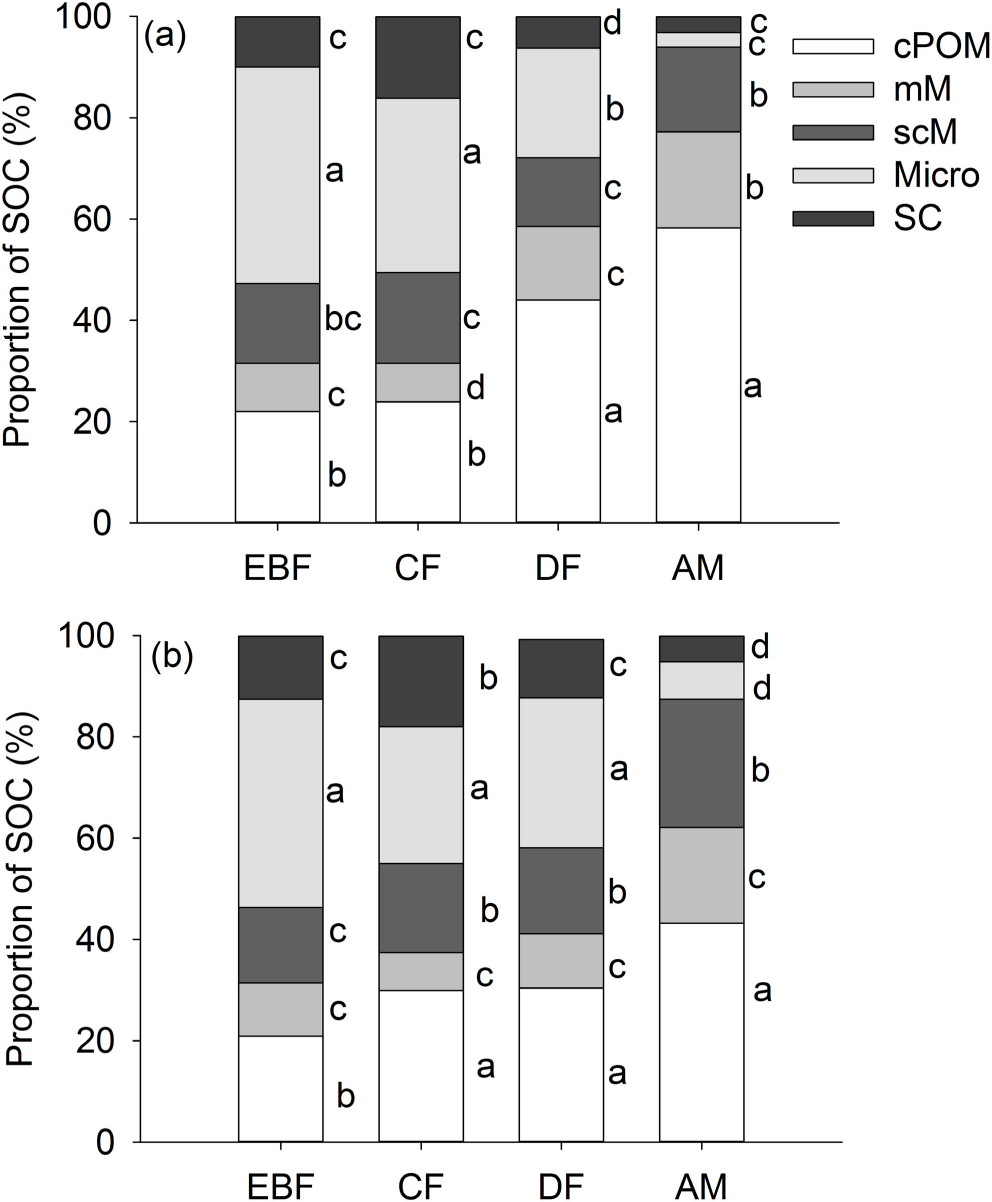
Distribution of organic carbon in coarse particulate organic matter (cPOM), microaggregates within the macroaggregates (mM), macroaggregate-occluded silt and clay particles (scM), microaggregates (Micro) and silt and clay (SC) particles of samples collected at an evergreen broad-leaf forest (EBF), coniferous forest (CF), sub-alpine dwarf forest (DF) and alpine meadow (AM) on Wuyi Mountain, China in the 0–10 cm layer (a) and 10–25 cm layer (b). Different letters for the sane vegetation indicate significant differences among the different size fractions of soil aggregates at P<0.05 (n = 4).

The distribution of SOC within different soil aggregate fractions varied with vegetation types (Fig 1). At both depths, SOC was mainly accumulated within the microaggregates in EBF and CF soils, but SOC was mainly accumulated within the coarse particulate organic matter fraction in DF and AM soils. SOC was accumulated the least in the silt and clay particles across all the four vegetation types at the 0–10 cm depth (Fig 1a). SOC accumulated in silt and clay particles was least in EBF and AM soils; however, the least SOC distributed in CF and DF soils was in microaggregates within macroaggregates at the 10–25 cm depth.

At the 0–10 cm depth, compared to coarse particulate organic matter fraction, mean C:N ratios in the silt and clay particles were decreased by 29%, 12%, 9.7% in CF, DF and EBF soils, respectively, but it was increased by 3.3% in AM soil (Table 3). At the 10–25 cm depth, the C: N ratios was decreased significantly in CF soil with decreasing aggregates size (Table 3).

### Correlation between soil aggregates and carbon accumulation

**Fig 2:**
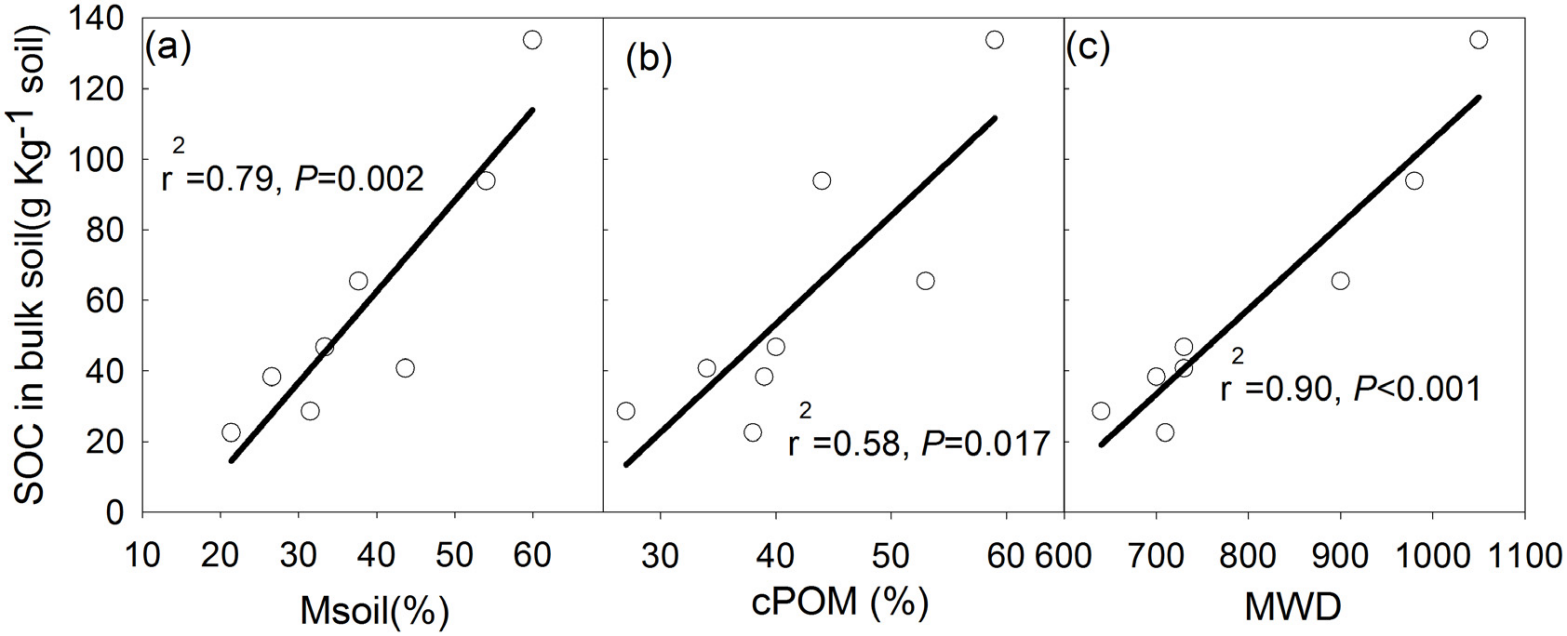
Relationship of SOC in bulk soil with soil moisture (a), coarse particulate organic matter content (b) and MWD (c).

The accumulation of C in bulk soils had a positive correlation with the percentage of SOC within the coarse particulate organic matter and microaggregates within the macroaggregates (r = 0.919 and 0.894 respectively). It had a negative relationship with the percentage of SOC within microaggregates and silt and clay particles (r = - 0.910 and 0.825, respectively) (Table 4). The accumulation of C in bulk soils had strong correlations with soil moisture, coarse particulate organic matter content and MWD (Fig 2). And it had no correlation with soil temperature and clay content (Table 4). Soil temperature exhibited a negative relationship with the percentage of C accumulated within the coarse particulate organic matter, and macroaggregate occluded silt and clay particles, while it demonstrated a positive relationship with the percentage of SOC in the microaggregate fraction (Table 4). MWD and soil moisture only showed no correlation with the percentage of SOC within macroaggregate-occluded silt and clay particles (Table 4). The amount of coarse particulate organic matter demonstrated strong correlations with the percentage of SOC in the coarse particulate organic matter, microaggregates within the macroaggregates and silt and clay particles fractions (Table 4). The amount of silt and clay particles showed strong correlations with percentage of SOC in the microaggregates within the macroaggregates and silt and clay particles fractions (Table 4).

**Table 4:** Correlation among bulk soil organic carbon (C_bulk_) with the percentage of coarse particulate organic matter (P_cPOM_), microaggregates within the macroaggregates (P_mM_), macroaggregate-occluded silt and clay particles (P_scM_), microaggregates (P_Micro_) and silt and clay particles (P_sc_) fractions in total organic C, soil temperature (T_soil_) and mean weight diameter (MWD) of the soil samples, silt and clay amount (SC), clay content, coarse particulate organic matter content (cPOM) and soil moisture (Msoil) collected at an evergreen broad-leaf forest (EBF), coniferous forest (CF), sub-alpine dwarf forest (DF) and alpine meadow (AM) on Wuyi Mountain, China (C_bulk_ was measured in mg g^-1^, Tsoil was measured in °C, MWD was measured in μm and other ones were measured in %).

	P_cPOM_	P_mM_	P_scM_	P_Micro_	P_sc_	Tsoil	Clay	SC
C_bulk_	0.919**	0.894**	0.367	-0.910**	-0.825*	-0.688	-0.114	0.575
Tsoil	-0.782*	-0.721*	-0.441	0.832*	0.648			
MWD	0.915**	0.965**	0.328	-0.885**	-0.914**			
Msoil	0.925**	0.849**	0.428	-0.944**	-0.779*			
cPOM	0.783*	0.783*	-0.095	-0.622	-0.869**			
SC	0.491	-0.724*	0.091	0.331	0.871**			

*Correlation is significant at the 0.05 level (2-tailed).

**Correlation is significant at the 0.01 level (2-tailed).

## Discussion

There has been an ongoing debate regarding the premise that SOC stocks increase with elevation. Based on the analysis of 2440 soil profiles in China, Xie, Sun [19] reported that elevation is the dominant factor that controls SOC accumulation in forest soils. However, Garten and Hanson [20] found no consistent trends for forest soil C inputs along elevation gradients, suggesting that altitudinal changes in soil C stocks and turnover times may be attributed to the differences in the organic matter decomposition of soils. Wang, Ruan [21], Shi, Wang [22] and Garten [23] pointed out that decreased soil temperatures with elevation tended to reduce soil respiration and the decomposition of SOC, thus resulting in altitudinal differences in C storage and SOC turnover. SOC in bulk soils showed no correlation with soil temperature (Table 4). And it had a stronger correlation with MWD (r^2^ = 0.90) than soil moisture (r^2^ = 0.79) (Fig 1), suggesting that soil aggregation may provide a degree of physical protection, and thus serve to prevent the decomposition of SOC. The positive correlation between bulk SOC and MWD was also reported by Smith, Tongway [24]. Previous studies have revealed that SOC decomposition rate in forest soils increased in the order: macroaggregates < microaggregates < silt and clay-sized complexes [5, 25, 26]. As aggregate size decreased, C: N ratios, plant litter components, and ^14^C labeled plant residues were also observed to decrease [27], whereas microbially-derived carbohydrates, and the ratio of alkyl/O-alkyl C increased [3]. However, significant decreases in C: N ratios with decreasing aggregate sizes occurred only in CF and DF soils at the 0–10 cm depth, and in CF soils at the 10-25cm depth. A large proportion of the soil organic matter within silt and clay particles(<53μm) was associated with “primary organomineral complexes” [28] and microbially decomposed [29, 30]. Hence, C: N ratios should be lowest within the silt and clay particles. However, the C: N ratios in the AM soils were similar for all the fractions (Table 3), suggesting that, due to the physical protection provided by silt and clay structures, parts of the labile organic materials were complexed with clay minerals, and thus became poorly bioavailable. Silt and clay particles played an important role in stabilizing SOC [31]. SOC stocks in the silt and clay fraction are mainly controlled by pedogenic properties such as clay content [32–34]. In this region, silt and clay content had a strong correlation with OC reserved in the silt and clay fraction (Table 4). However, coarse particulate organic matter content also correlated well with OC reserved in the silt and clay fraction. Moreover, it showed good correlations with OC content in bulk soil, microaggregates within macroaggregates, macroaggregate-occluded silt and clay particles, and microaggregates (Table 4, Fig 2). Compared to silt and clay particles content, coarse particulate organic matter content was a better index to represent organic carbon distribution in bulk soil and different soil aggregate fractions.

When fresh plant materials (litter or roots) enter soils, they become a source of C for microbial activity. During the utilization process, soil fungi and other soil microorganisms produce mucilage, resulting in the formation of macroaggregates that encapsulate the cPOM fractions [35]. Subsequently, the cPOM fractions are further decomposed and fragmented into fine POM, where fine POM and associated mucilage become encrusted with minerals to form the stabilized organic cores of microaggregates within the macroaggregates [35, 36]. As microaggregates are less accessible to microbes, the generation of binding agents are reduced, and the stability of microaggregates is decreased. Following the breakdown of microaggregates, mineral crusts impregnated with microbial byproducts are released and facilitate the stabilization of SOC with silt and clay particles [30, 37–40]. Therefore, high decomposition rates of litter and SOC at low altitudes [22, 41] may result in high macroaggregate turnover rates. Consequently, the percentage of macroaggregates increased from EBF to AM (Table 2). Correspondingly, the dominant SOC reservoir changed from microaggreages and silt and clay particles to macroaggregates (Fig 1).

Macroaggregates are bound together by transient and temporary binding agents (primarily polysaccharides, and roots and hyphae, respectively) [25]. Microaggregates, silt and clay particles are held together by persistent binding agents (e.g., strongly sorbed organic polymers, oxides, and polyvalent cations). Temporary binding agents only last several days; however, persistent agents could remain for decades. Macroaggregates are more vulnerable to environmental change than microaggregates and silt and clay particles [42]. It is silt and clay, rather than macroaggregates, associated organic matter that is critical for medium and longer term organic matter turnover [43]. Macroaggregates accounted for 57–93% of total soil aggregates, which is consistent with the observation that abundant macroaggregates (53–91%) were present in forest soils [44–46]. Most SOC was accumulated in macroaggregates, indicating that organic materials were the major binding agent for aggregates in these soils, and that the SOC accumulated within the macroaggregates possessed limited longevity. Compared to other two fractions, coarse particulate organic matter fractions contributed most to SOC in bulk soils (Fig 1). It accounted for 34% of the total SOC. This is slightly higher than the reported value by Gregorich, Beare [47], that coarse particulate organic matter fractions accounted for 27% of the total SOC of 13 forest soils. At the 0–10 cm depth, 58% SOC was in coarse particulate organic matter fraction in AM soil (Fig 1). The percentage was 2.6 times higher than that in the EBF soils (22%) (Fig 1). The percentage of the SOC accumulated within the silt and clay particles in AM soils was only 33% of the amount that was resident in the EBF soils (Fig 1). With increasing aggregation rate, the larger labile pool of higher altitude SOC may be at a higher risk of decomposition when exposed to warming climate conditions, and hence, SOC losses may be disproportionately higher in the more aggregated soils.

## Conclusions

The concentrations of SOC in different soil aggregate fractions and bulk soils increased with increasing soil aggregation rate. From EBF to AM, with increasing soil aggregation rate, the accumulation of SOC was shifted from the microaggregate to the macroaggregate fraction, particularly the coarse particulate organic matter fraction. Coarse particulate organic matter content, better than silt and clay particles content, represented organic carbon accumulation in bulk soil and different soil aggregate fractions. Due to the protection provided by soil aggregation and organic matter sorption to the mineral surface (silt and clay particles), labile organic carbon was also present in silt and clay particles. Because of the intrinsic liability and vulnerability of soil macroaggregates, a larger percentage of SOC stored in the macroaggregate fraction may indicate a higher risk of carbon loss when subjected to the warming climate. With increasing soil aggregation rate, the SOC loss rates in higher altitude soils are likely greater when subjected to temperature increases, in contrast low altitude soils. Further lab or field experimentations with modified temperatures are warranted to elucidate the SOC decomposition rates in different size fractions of soil aggregates.

## References

[1] Smith P, Fang CM, Dawson JJC, Moncrieff JB. Impact of Global Warming on Soil Organic Carbon. Adv Agron. 2008;97:1–43. 10.1016/S0065-2113(07)00001-6

[2] Davidson EA, Janssens IA. Temperature sensitivity of soil carbon decomposition and feedbacks to climate change. Nature. 2006;440(7081):165–73. Epub 2006/03/10. 10.1038/nature04514 . 16525463

[3] Steffens M, Kölbl A, Kögel-Knabner I. Alteration of soil organic matter pools and aggregation in semi-arid steppe topsoils as driven by organic matter input. Eur J Soil Sci. 2009;60(2):198–212. 10.1111/j.1365-2389.2008.01104.x

[4] Six J, Callewaert P, Lenders S, De Gryze S, Morris SJ, Gregorich EG, et al Measuring and understanding carbon storage in Afforested Soils by Physical Fractionation. Soil Sci. 2002;66:1981–7. 10.2136/sssaj2002.1981

[5] Six J, Bossuyt H, Degryze S, Denef K. A history of research on the link between (micro)aggregates, soil biota, and soil organic matter dynamics. Soil Till Res. 2004;79(1):7–31. 10.1016/j.still.2004.03.008

[6] Pulleman MM, Six J, van Breemen N, Jongmans AG. Soil organic matter distribution and microaggregate characteristics as affected by agricultural management and earthworm activity. Eur J Soil Sci. 2005;56(4):453–67. 10.1111/j.1365-2389.2004.00696.x

[7] Bu XL, Ruan HH, Wang LM, Ma WB, Ding JM, Yu XN. Soil organic matter in density fractions as related to vegetation changes along an altitude gradient in the Wuyi Mountains, southeastern China. Applied Soil Ecology. 2012;52:42–7. 10.1016/j.apsoil.2011.10.005 WOS:000298975100005.

[8] Bissonnais YL. Aggregate stability and assessment of soil crustability and erodibility: I. Theory and methodology. Eur J Soil Sci. 1996;47(4):425–37. 10.1111/j.1365-2389.1996.tb01843.x

[9] Rasmussen C, Torn MS, Southard RJ. Mineral assemblage and aggregates control carbon dynamics in a California conifer forest. Soil Sci Soc Am J. 2005;69(6):1711–21. 10.2136/sssaj2005.0040 WOS:000233223500005.

[10] Birdsey RA, Platinga AJ, Heath LS. Past and prospective carbon storage in United States forests. Forest Ecol Manag. 1993;58(1–2):33–40. 10.1016/0378-1127(93)90129-B

[11] Zhou XY, Zhang CY, Guo GF. Effects of climate change on forest soil organic carbon storage: a review. The journal of applied ecology. 2010;21(7):1867–74 (in Chinese with English abstract). MEDLINE:. 20879549

[12] von Luetzow M, Koegel-Knabner I. Temperature sensitivity of soil organic matter decomposition-what do we know? Biol Fert Soils. 2009;46(1):1–15. 10.1007/s00374-009-0413-8 WOS:000271527400001.

[13] Plante AF, Fernandez JM, Haddix ML, Steinweg JM, Conant RT. Biological, chemical and thermal indices of soil organic matter stability in four grassland soils. Soil Biol Biochem. 2011;43(5):1051–8. 10.1016/j.soilbio.2011.01.024 WOS:000289219500023.

[14] Djukic I, Zehetner F, Tatzber M, Gerzabek MH. Soil organic-matter stocks and characteristics along an Alpine elevation gradient. J Soil Sci Plant Nut. 2010;173(1):30–8. 10.1002/jpln.200900027 WOS:000275220000004.

[15] Griffiths RP, Madritch MD, Swanson AK. The effects of topography on forest soil characteristics in the Oregon Cascade Mountains (USA): Implications for the effects of climate change on soil properties. Forest Ecol Manag. 2009;257(1):1–7. 10.1016/j.foreco.2008.08.010

[16] Xu XF, Chen YQ, Wang JS, Fang YH, Quan W, Ruan HH, et al Variations of soil labile organic carbon along an altitude gradient in Wuyi Mountain. The Journal of Applied Ecology. 2008;19(3):539–44. MEDLINE:. 18533522

[17] Bu XL, Ding JM, Wang LM, Yu XN, Huang W, Ruan HH. Biodegradation and chemical characteristics of hot-water extractable organic matter from soils under four different vegetation types in the Wuyi Mountains, southeastern China. Eur J Soil Biol. 2011;47(2):102–7. 10.1016/j.ejsobi.2010.11.009

[18] Bu XL, Wang LM, Ma WB, Yu XN, McDowell WH, Ruan HH. Spectroscopic characterization of hot-water extractable organic matter from soils under four different vegetation types along an elevation gradient in the Wuyi Mountains. Geoderma. 2010;159(1–2):139–46. 10.1016/j.geoderma.2010.07.005

[19] Xie XL, Sun B, Zhou HZ, Li AB. Soil organic carbon storage in China. Pedosphere. 2004;14(4):491–500. WOS:000224774000010.

[20] Garten CT Jr, Hanson PJ. Measured forest soil C stocks and estimated turnover times along an elevation gradient. Geoderma. 2006;136(1–2):342–52. 10.1016/j.geoderma.2006.03.049 WOS:000242837500030.

[21] Wang SJ, Ruan HH, Han Y. Effects of microclimate, litter type, and mesh size on leaf litter decomposition along an elevation gradient in the Wuyi Mountains, China. Ecological Research. 2010;25(6):1113–20. 10.1007/s11284-010-0736-9 WOS:000284422200007.

[22] Shi Z, Wang JS, He R, Wang GB, Fang YH, Xu ZK, et al Seasonal variation and temperature sensitivity of soil respiration under different plant communities along an elevation gradient in Wuyi Mountains of China. Chinese Journal of Applied Ecology. 2008;19(11):2357–63 (in Chinese with English abstract). 19238832

[23] Garten CT Jr. Comparison of forest soil carbon dynamics at five sites along a latitudinal gradient. Geoderma. 2011;167–68:30–40. 10.1016/j.geoderma.2011.08.007 WOS:000298029000005.

[24] Smith R, Tongway D, Tighe M, Reid N. When does organic carbon induce aggregate stability in vertosols? Agriculture Ecosystems & Environment. 2015;201:92–100. 10.1016/j.agee.2014.12.002 WOS:000350190100010.

[25] Christensen BT. Physical fractionation of soil and structural and functional complexity in organic matter turnover. Eur J Soil Sci. 2001;52(3):345–53. 10.1046/j.1365-2389.2001.00417.x ISI:000170903600001.

[26] Gupta VVSR, Germida JJ. Soil aggregation: Influence on microbial biomass and implications for biological processes. Soil Biology & Biochemistry. 2015;80:A3–A9. WOS:000346545800002.

[27] Gregorich EG, Beare MH, Stoklas U, St-Georges P. Biodegradability of soluble organic matter in maize-cropped soils. Geoderma. 2003;113(3–4):237–52. 10.1016/s0016-7061(02)00363-4

[28] Virto I, Barre P, Chenu C. Microaggregation and organic matter storage at the silt-size scale. Geoderma. 2008;146(1–2):326–35. 10.1016/j.geoderma.2008.05.021 WOS:000258995600038.

[29] Grandy AS, Neff JC. Molecular C dynamics downstream: The biochemical decomposition sequence and its impact on soil organic matter structure and function. Sci Total Environ 2008;404(2–3):297–307. 10.1016/j.scitotenv.2007.11.013 ISI:000260701900011. 18190951

[30] Spielvogel S, Prietzel J, Kogel-Knabner I. Soil organic matter stabilization in acidic forest soils is preferential and soil type-specific. Eur J Soil Sci. 2008;59(4):674–92. 10.1111/j.1365-2389.2008.01030.x ISI:000257550900007.

[31] Mao DH, Wang ZM, Li L, Miao ZH, Ma WH, Song CC, et al Soil organic carbon in the Sanjiang Plain of China: storage, distribution and controlling factors. Biogeosciences. 2015;12(6):1635–45. 10.5194/bg-12-1635-2015 WOS:000352112900001.

[32] Hassink J. The capacity of soils to preserve organic C and N by their association with clay and silt particles. Plant and Soil. 1997;191(1):77–87. 10.1023/A:1004213929699 WOS:A1997XP69700008.

[33] Gruneberg E, Schoning I, Hessenmoller D, Schulze ED, Weisser WW. Organic layer and clay content control soil organic carbon stocks in density fractions of differently managed German beech forests. Forest Ecol Manag. 2013;303:1–10. 10.1016/j.foreco.2013.03.014 WOS:000321405600001.

[34] Wiesmeier M, Munro S, Barthold F, Steffens M, Schad P, Kogel-Knabner I. Carbon storage capacity of semi-arid grassland soils and sequestration potentials in northern China. Global Change Biol. 2015;21(10):3836–45. 10.1111/gcb.12957 WOS:000360994500023. 25916410

[35] Six J, Elliott ET, Paustian K. Soil structure and soil organic matter: II. A normalized stability index and the effect of mineralogy. Soil Sci Soc Am J. 2000;64(3):1042–9.

[36] Angers DA, Recous S, Aita C. Fate of carbon and nitrogen in water-stable aggregates during decomposition of ^13^C^15^N-labelled wheat straw in situ. Eur J Soil Sci. 1997;48(2):295–300. 10.1111/j.1365-2389.1997.tb00549.x

[37] Golchin J, Nelson S. Source removal strategy development for manufactured gas plant sites. P Haz Wast Remed. 1994:187–97. ISI:A1994BD28X00012.

[38] Chenu C, Plante AF. Clay-sized organo-mineral complexes in a cultivation chronosequence: revisiting the concept of the 'primary organo-mineral complex'. Eur J Soil Sci. 2006;57(4):596–607. 10.1111/j.1365-2389.2006.00834.x ISI:000238486100017.

[39] Wan J, Tyliszczak T, Tokunaga TK. Organic carbon distribution, speciation, and elemental correlations within soil micro aggregates: Applications of STXM and NEXAFS spectroscopy. Geochim Cosmochim Ac. 2007;71(22):5439–49. 10.1016/j.gca.2007.07.030 ISI:000251052100011.

[40] Bachmann J, Guggenberger G, Baumgartl T, Ellerbrock RH, Urbanek E, Goebel MO, et al Physical carbon-sequestration mechanisms under special consideration of soil wettability. Journal of Plant Nutrition and Soil Science-Zeitschrift Fur Pflanzenernahrung Und Bodenkunde. 2008;171(1):14–26. ISI:000253431000002.

[41] Wang S, Ruan H, Wang B. Effects of soil microarthropods on plant litter decomposition across an elevation gradient in the Wuyi Mountains. Soil Biology and Biochemistry. 2009;41(5):891–7. 10.1016/j.soilbio.2008.12.016

[42] Bronick CJ, Lal R. Soil structure and management: a review. Geoderma. 2005;124(1–2):3–22. 10.1016/j.geoderma.2004.03.005 WOS:000226150900001.

[43] Christensen BT. Carbon and Nitrogen in Particle Size Fractions Isolated from Danish Arable Soils by Ultrasonic Dispersion and Gravity-Sedimentation. Acta Agr Scand. 1985;35(2):175–87. 10.1080/00015128509435773

[44] Shrestha BM, Singh BR, Sitaula BK, Lal R, Bajracharya RM. Soil aggregate- and particle-associated organic carbon under different land uses in Nepal. Soil Sci Soc Am J. 2007;71(4):1194–203. 10.2136/sssaj2006.0405 WOS:000248103200013.

[45] Schwendenmann L, Pendall E. Effects of forest conversion into grassland on soil aggregate structure and carbon storage in Panama: evidence from soil carbon fractionation and stable isotopes. Plant Soil 2006;288(1–2):217–32. 10.1007/s11104-006-9109-0 WOS:000243001400017.

[46] Singh S, Singh JS. Water-stable aggregates and associated organic matter in forest, savanna, and cropland soils of a seasonally dry tropical region, India. Biol Fert Soils. 1996;22(1–2):76–82. 10.1007/BF00384436

[47] Gregorich EG, Beare MH, McKim UF, Skjemstad JO. Chemical and Biological Characteristics of Physically Uncomplexed Organic Matter. Soil Sci Soc Am J. 2006;70(3):975 10.2136/sssaj2005.0116

[48] He JY, Lan SR, Liu CD, Li LH. Wuyishan Research In: He J.Y., editor, Natural resource Ser., Xiamen University Press, Xiamen p. 39–117. (In Chinese). 1994.

[49] He R, Wang GB, Wang JS, Xu BF, Wang KJ, Fang YH, et al Seasonal variation and its main affecting factors of soil microbial biomass under different vegetations along an elevation gradient in Wuyi Mountains of China. Chinese Journal of Ecology 2009;28(3):394–9 (in Chinese with English abstract).

[50] Chen JF. Genetic characteristics and taxonomic classification of soils in the Mountain Wuyi. Chinese Journal of Soil Science. 2000;31(3):97–101 (in Chinese with English abstract).

